# Changes in management of FETO-treated infants with severe CDH

**DOI:** 10.1007/s00431-026-07526-w

**Published:** 2026-10-24

**Authors:** Eleanor Jeffreys, Allan Jenkinson, Rania Kronfli, Jennifer Billington, Ravindra Bhat, Mahesh Nanjundappa, Kamal Ali, Ancuta Muntean, Katharine Harman, Adam Lawton, Ghalia Ashoor, Kypros Nicolaides, Theodore Dassios, Anne Greenough, Christopher Harris

**Affiliations:** 1 Department of Women and Children’s Health, School of Life Course Sciences, Faculty of Life Sciences and Medicine, King’s College London, London, UK; 2 Neonatal Intensive Care Centre, King’s College Hospital NHS Foundation Trust, 4, Floor Golden Jubilee Wing, Denmark Hill, London, SE5 9RS UK; 3 Department of Paediatric Surgery, King’s College Hospital NHS Foundation Trust, London, UK; 4 Fetal Medicine Research Institute, King’s College Hospital, London, UK; 5 Neonatal Intensive Care Department, King Abdulaziz Medical City, Riyadh, Saudi Arabia

**Keywords:** Congenital diaphragmatic hernia, Fetoscopic endoluminal tracheal occlusion, Neonatal, Laterality, Respiratory

## Abstract

This study aims to evaluate changes in outcomes and management of infants with severe congenital diaphragmatic hernia (CDH) undergoing fetoscopic endoluminal tracheal occlusion (FETO) across two eras, and to compare outcomes by side of defect over the last decade. Retrospective review of liveborn infants with severe CDH (observed to expected lung to head ratio [O/E LHR] < 0.25 left-sided; < 0.45 right-sided) who underwent FETO at King’s College Hospital, London, UK, and survived to delivery. Fifty-eight infants were included across two 5-year periods (2002–2007 and 2020–2025). Survival improved from 44.1% to 70.8% (*p* = 0.044). Right-sided defects were more common in the second epoch (9% versus 54%, *p* < 0.001). All infants in the second era had echocardiography-guided inotropic and ventilatory management, which was not universally available earlier. In the second epoch surgical repair occurred later (4.0 versus 6.5 days, *p* = 0.018), repair by two-stage abdominal closure (laparostomy) increased from 0% to 77.8% (*p* < 0.001) and recurrence fell from 33 to 0% (*p* = 0.042). Mechanical ventilation, oxygen therapy and admission were longer latterly. In thirty-one infants delivered between 2015 and 2025, preterm prelabour rupture of membranes (PPROM) occurred in 67% of left-versus 92% of right-sided CDH, but at an earlier gestational age in left (*p* = 0.032). Left CDH infants had longer duration of mechanical ventilation (*p* = 0.04) and hospital stay (*p* = 0.006) with no mortality difference (exploratory). *Conclusion*: Survival of FETO-treated severe CDH infants improved from 44 to 71% alongside later repair, staged abdominal closure and echocardiography-guided management. Left-sided defects had greater respiratory morbidity and longer admission than right-sided defects without changing survival.

**Table:** 

**What is Known**: • FETO improves survival in severe left-sided CDH, but postnatal management has changed substantially since its introduction.	
**What is New**: • Survival of FETO-treated severe CDH rose from 44 to 71% over two decades despite more right-sided defects. Repair occurred later, following haemodynamic and respiratory stabilisation (as guided by echocardiography) with staged abdominal closure in 77.8% of recent repairs; recurrence decreased from 33 to 0%. • Left-sided CDH infants had greater respiratory morbidity and length of stay than right-sided CDH infants, without a significant difference in mortality.

**What is Known**:

• FETO improves survival in severe left-sided CDH, but postnatal management has changed substantially since its introduction.

**What is New**:

• Survival of FETO-treated severe CDH rose from 44 to 71% over two decades despite more right-sided defects. Repair occurred later, following haemodynamic and respiratory stabilisation (as guided by echocardiography) with staged abdominal closure in 77.8% of recent repairs; recurrence decreased from 33 to 0%.

• Left-sided CDH infants had greater respiratory morbidity and length of stay than right-sided CDH infants, without a significant difference in mortality.

## Introduction

Congenital diaphragmatic hernia (CDH) occurs in approximately 1 in 2000–4000 births in the United Kingdom [1, 2] and is associated with a mortality of 25 to 30% [1, 3]. The risk of dying due to CDH can be assessed by measuring the observed to expected lung to head ratio (O/E LHR). The mortality is highest in those with the lowest O/E LHR and a severe defect is defined as an O/E LHR as less than 0.25 for left sided hernias [4] and less than 0.45 for right sided defects [5]. Severe CDH differs from moderate and mild disease because the reduced fetal lung size reflects more severe pulmonary hypoplasia, altered pulmonary vascular modelling and cardiac ventricular dysfunction, which is associated with worse postnatal respiratory outcomes and survival [4, 6].

Tracheal occlusion in animal models of CDH has been shown to improve lung development [7]. The development of minimally invasive fetoscopic techniques in humans has enabled a percutaneous, endoluminal approach to tracheal occlusion. In the ‘Tracheal Occlusion To Accelerate Lung Growth’ (TOTAL) trial, fetoscopic endoluminal tracheal occlusion (FETO) was achieved by placing an inflatable balloon in the fetal trachea between 27 and 29 weeks of gestation [8]. This multicentred trial recruited severe left-sided defects and demonstrated significantly improved survival to discharge (40% versus 15%), albeit with a higher incidence of preterm birth [9]. Recruitment to the TOTAL trial ended in 2020.

Right-sided CDH lesions are less prevalent than left-sided ones but account for 15–20% of cases. In 2007 Jani et al. reported survival rates for left sided CDH infants with O/E LHR less than 0.25, to be 30%, which decreased to 15% with liver involvement. For right sided CDH with an O/E LHR less than 0.45, the survival rates were negligible [4]. The TOTAL trial examined only infants with left sided lesions although since 2020 fetuses with right sided defects are eligible for FETO.

During the FETO era, there have been substantial advances in postnatal surgical and medical management of infants with CDH. This includes standardised delivery room protocols, gentle ventilation strategies, refined use of extracorporeal membrane oxygenation, improved management of pulmonary hypertension, and optimization of timing and technique of surgical repair [10–12]. Collectively, these changes could have impacted on clinical outcomes.

King's College Hospital NHS Foundation Trust (KCH) is a high volume FETO centre, performing more than 36 fetoscopies per year as defined by the TOTAL trial [9], within a tertiary neonatal intensive care setting. The aims of our study were to document if there were changes in outcomes for infants with severe CDH who underwent FETO over two time periods and whether this related to changes in management. Furthermore, we wished to determine if outcomes in infants who recently had FETO differed by side of defect.

## Materials and methods

A retrospective review of the medical records for liveborn infants diagnosed with severe CDH, who underwent FETO at KCH and survived to delivery was conducted. Severe CDH was defined as observed to expected lung head ratio (O/E LHR) < 0.25 for left-sided and < 0.45 for right-sided defects. We undertook two analyses: temporal comparison of two treatment eras and laterality comparison in a contemporary cohort. In the temporal comparison, the first (2002–2007) and second epoch (2020–2025) were selected to compare FETO CDH outcomes. The initial epoch was the first 5 years following the initiation of FETO. The second epoch was the most recent 5 years, commencing at the end of the TOTAL trial period. These timeframes were chosen because right-sided defects only became eligible for FETO from 2020 following conclusion of the TOTAL trial and practice during the trial was governed by the trial protocol rather than routine care. For laterality we compared FETO outcomes by side of defect between 2015 and 2025. All infants meeting the eligibility criteria during each study period were included and the study size was determined by the number of eligible FETO-treated infants at our centre. The study was registered with the clinical governance and audit department of KCH with reference number: CH202. The Health Research Authority Toolkit of the National Health Service, United Kingdom, verified that the study would not need regulatory approval by a research ethics committee.

After hospital discharge, all patients were followed in paediatric surgical and neonatal clinics at 3–6-month intervals for at least 1 year. If no active issues were noted, patients were seen annually at the paediatric surgical clinic, during which a routine screening chest radiograph was performed. More recently, follow-up has taken place in the joint CDH multidisciplinary team clinic. Median follow-up duration was not available, and some patients in the later cohort will have had less than 1 year of follow-up.

Infants with concomitant congenital abnormalities other than CDH were excluded. Data collected included: gestational age (GA); birth weight (BW); observed to expected lung to head ratio prior to FETO (O/E LHR); gestational age at the time of FETO; preterm prelabour rupture of membranes (PPROM) defined as loss of fetal membranes for more than 48 h prior to commencement of labour (spontaneous and not occurring during fetoscopy); antenatal steroid administration; mode of delivery; sex; age of infant at CDH repair; left versus right sided defect.

From 2016, clinical stabilisation of an infant with CDH prior to surgery was according to the CDH EURO Consortium Consensus. The criteria were as follows: a mean arterial blood pressure normal for gestation; preductal saturation levels of 85–95% on FiO2 < 50%; lactate below 3 mmol/l and a urine output more than 1 ml/kg/h [11].

Outcome data collected included: mortality (defined as death during neonatal stay); requirement for extracorporeal membrane oxygenation (ECMO); length of mechanical ventilation; length of supplementary oxygen requirement; supplementary oxygen at 28 days; supplementary oxygen at discharge; length of admission; echocardiography diagnosed pulmonary hypertension. Surgical outcome data included: age at CDH repair; repair via laparostomy versus laparotomy (laparostomy defined as intentionally leaving the abdominal cavity open with delayed fascial closure once the infant was stable); repair using a patch; CDH recurrence (defined as re-herniation of abdominal contents through the diaphragmatic repair, identified on routine screening chest radiograph or on symptomatic presentation). Additionally, feeding outcomes were collected: anti-reflux medication, tube feeding, gastrostomy and need for fundoplication surgery.

### Analysis

Data were analysed using SPSS (IBM Corp., Armonk, NY, USA; version 31.0.0.0). Data were examined for normality using the Shapiro–Wilk test and found to be non-normally distributed and hence reported as median (interquartile range). Categorical data were analysed for statistical significance using the chi-square test and Fisher’s Exact Test (where the expected cell count was less than five). Non-normally distributed continuous data were analysed for statistical significance using the Mann–Whitney *U* test. The Pearson chi-square test was pre-specified and, as all expected cell frequencies exceeded five, no continuity correction was applied. Effect sizes (phi coefficient and odds ratio with 95% confidence interval) were calculated for the comparison of survival. Due to the small number of deaths (*n* = 26), the data did not allow a reliable multivariable adjusted analysis. The laterality comparison was considered exploratory. Several comparisons yielded *p*-values close to 0.05 and no adjustment was made for multiple comparisons. To limit selection bias and heterogeneity, we restricted the cohorts to liveborn infants with isolated severe CDH treated with FETO at KCH, defined eligibility by prespecified O/E LHR thresholds, and used the same outcome definitions for both eras. The study is reported in accordance with the STROBE guidelines for observational cohort studies.

## Results

**Fig. 1:**
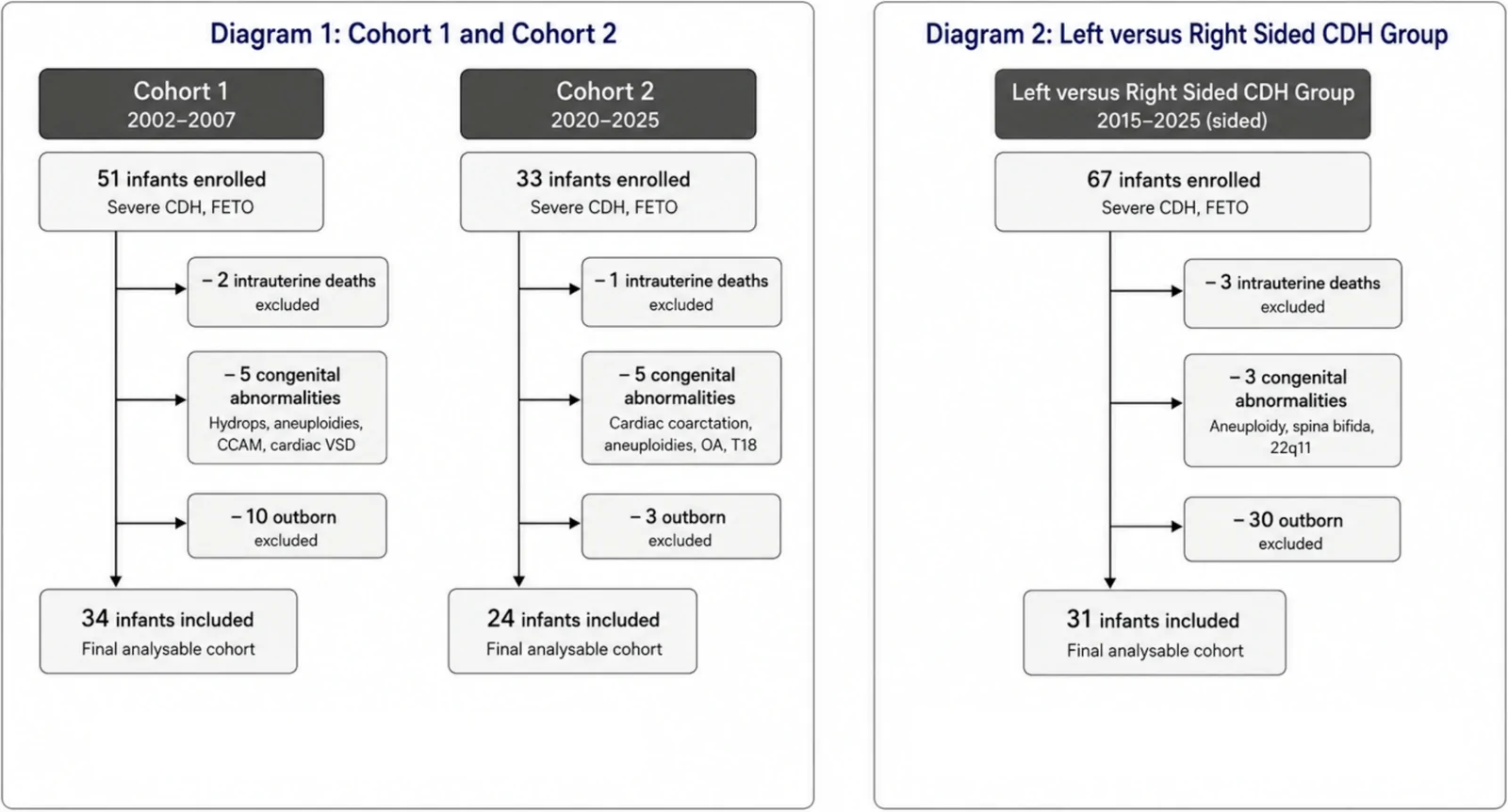
Cohort flow diagram showing infant inclusion

Fifty-one infants were identified as having severe CDH and underwent FETO in the 2002–2007 period. Seventeen were excluded with two intrauterine deaths (3.9%), five due to additional congenital abnormalities (hydrops, aneuploidies, CCAM, cardiac VSD) and 10 infants outborn and therefore without morbidity or mortality data. The final cohort included 34 infants. Thirty-three infants were identified in the 2020–2025 period. Nine infants were excluded resulting in a final cohort of 24 infants (Fig. 1). Exclusions were: one intrauterine death (3.0%), five with additional congenital abnormalities (cardiac coarctation, aneuploidies, oesophageal atresia, trisomy 18) and three who were outborn.

For the 2015–2025 data, the initial cohort included 67 patients. 36 were excluded: three intrauterine deaths (4.5%), three exclusions for congenital abnormalities (aneuploidy, spina bifida, 22q11 deletion) and thirty outborn infants. The final cohort included 31 infants: 18 with left sided defects and 13 with right sided.

Intrauterine deaths were uncommon ranging from 3.0 to 4.5% across the three groups. The larger proportion excluded in the 2015–2025 cohort was driven by outborn infants (referrals delivered outside KCH) rather than by antenatal or intrauterine loss.

The 2015–2025 laterality cohort (*n* = 31: 18 left, 13 right) and the 2020–2025s epoch (*n* = 24: 11 left, 13 non-left) overlapped. The second epoch included one infant with bilateral CDH, who survived; this infant was included in the non-left group for the epoch comparison but was not included in the laterality analysis, as no sided comparison was possible. The two cohorts differed by seven additional left-sided infants and one additional right-sided infant treated between 2015 and 2019. The right-sided infant did not survive. Of the 18 left-sided infants in the 2015–2025 cohort, nine died (Table 4) and nine survived; eight of these survivors were among the 11 left-sided infants in the 2020–2025 epoch, so the eight infants treated in 2015–2019 contributed one survivor.

### Comparison of the first and second cohort

The gestational age (*p* = 0.962) and birth weight (*p* = 0.931) were comparable between the two cohorts. There were more right sided defects in the later cohort (*p* < 0.001). The O/E LHR was higher in the more recent cohort (*p* = 0.018) (Table 1).

**Table 1:** Clinical characteristics and demographic data. Data are presented as *n*(%) and median (interquartile range). In the 2020–2025 epoch, the figures for right-sided defects include one infant with bilateral CDH, who survived. The asterisk (*) indicates where count < 5 and Fisher’s Exact Test used. Denominators shown where there was data unavailability—repair via patch or laparostomy was not known for one patient in epoch one and was not known for the patient transferred out for ECMO in epoch 2; out-of-area status was available for 33 patients in epoch one

	2002–2007 (*n* = 34)	2020–2025 (*n* = 24)	*p* value
GA (weeks)	34.9 (32.08–36.33)	34.8 (32.45–35.98)	0.962
BW (grams)	2319 (1607.50–2738.00)	2262 (1850.00–2786.25)	0.931
Male sex	20 (59)	20 (83)	0.047
Left sided	31 (91)	11 (46)	< 0.001
US O/E LHR	0.16 (0.14–0.19)	0.21 (0.15–0.24)	0.018
US O/E LHR left sided defects	0.16 (0.13–0.19)	0.19 (0.13–0.23)	0.125
US O/E LHR right sided defects	0.31 (0.16–0.31)	0.21 (0.20–0.25)	0.309
GA FETO (weeks)	26.3 (25.25–27.55)	26.7 (26.10–27.29)	0.081
Antenatal steroids	14 (41.2)	18 (75)	0.011
Preterm prelabour rupture of membranes	24 (71)	19 (79)	0.462
GA PPROM (weeks)	33.1 (27.93–34.23)	33.9 (31.22–35.00)	0.117
Delivery by caesarean section	10 (29)	7 (29)	0.98
Balloon out at delivery	23 (68)	17 (71)	0.796
Death due to inability to deflate FETO balloon	3 (8.8)	0 (0)	0.26*
Day of repair	4.0 (1.0–6.0)	6.5 (4.75–8.0)	0.018
Repair using patch	14/19 (74)	17/18 (94)	0.18*
Repair with laparostomy	0/19 (0.0)	14/18 (77.8)	< 0.001*
Infants treated and born at KCH from out of area	30/33 (90.9%)	22/24 (91.7%)	1.0*

Survival of left sided CDH infants in the first epoch was 13/31 (41.9%) compared to 8/11 (72.7%) in the second epoch, *p* = 0.08. Survival of right sided CDH infants (non-left sided infants in the second epoch, including one infant with bilateral CDH who survived) in the first epoch was 2/3 (66.7%) compared to 9/13 (69.2%) in epoch two, *p* = 1.0 (Fisher’s exact test).

Repair occurred later in the second epoch (*p* = 0.018). The use of laparostomy and staged closure was newly introduced and performed in 77.8% of cases in the second epoch compared to none in the first (*p* < 0.001) (Table 1).

The proportion of infants undergoing surgical repair was 58.8% in the first epoch and 79.2% in the second (*p* = 0.104). Mortality before repair was 41.2% and 20.8% (*p* = 0.104), and mortality after repair was 14.7% and 8.3% (*p* = 0.688), respectively (Table 2). Duration of mechanical ventilation and oxygen therapy were calculated for survivors to discharge only.

**Table 2:** Outcome data. Data are presented as *n* (%) and median (interquartile range). The asterisk (*) indicates where count < 5 and Fisher’s Exact Test used. Of note, in the second epoch, one patient was transferred out for ECMO and one patient transferred out for ENT review and bronchoscopy; survival data but no further outcome data was known for these patients. Duration of mechanical ventilation and oxygen therapy were calculated for survivors to discharge only. Oxygen at 28 days and at discharge, anti-reflux medication, tube feeding, gastrostomy and fundoplication were reported for survivors only (*n* = 15 in each epoch), excluding the two infants transferred to other centres

	2002–2007 (*n* = 34)	2020–2025 (*n* = 24)	*p* value
Survival	15 (44)	17 (70.8)	0.044
Mortality prior to repair	14 (41.2)	5 (20.8)	0.104
Mortality post repair	5 (14.7)	2 (8.3)	0.688*
Proportion of infants undergoing surgical repair	20 (58.8)	19 (79.2)	0.104
Recurrence of defect	5/15 (33)	0/15 (0.0)	0.042*
Duration MV (days)	11.0 (7.750–21.25)	21.0 (15.5–29.0)	0.026
Duration O2 therapy (days)	11.5 (9.5–25.75)	41.0 (35.0–54.00)	0.001
Length admission (days)	44.0 (32.0–57.0)	61.0 (44.0–83.0)	0.049
Age full feeds (days)	27.5 (20.50–39.25)	35.2 (26.00–42.00)	0.478
O2 at 28 days	3/15 (20.0)	13/15 (86.7)	0.001*
O2 at discharge	2/15 (13.3)	2/15 (13.3)	1.0*
Anti-reflux medication	3/15 (20)	13/15 (87)	< 0.001
Tube feeding	5/15 (33)	7/15 (47)	0.71*
Gastrostomy	2/15 (13.3)	3/15 (20.0)	1.0*
Fundoplication	2/15 (13.3)	1/15 (6.7)	1.0*

Survival was significantly increased to 70.8% in the second epoch compared to 44.1% in the first. *χ*^2^(1) = 4.06, *p* = 0.044, *φ* = 0.26; OR 3.08, 95% CI 1.01 to 9.34. Recurrence of the diaphragmatic hernia decreased from 33% to zero (*p* = 0.042). Duration of mechanical ventilation and supplemental oxygen requirements was significantly increased in the second epoch compared to the first (*p* = 0.026 and *p* = 0.001 respectively) as was the length of hospital stay (*p* = 0.049) (Table 2).

The earlier cohort had no infants, 0/34 (0%), transferred out for ECMO as compared to one infant, 1/24 (4.2%) in the later cohort. The second epoch also had one patient transferred out for ENT review and bronchoscopy. Survival data but no further outcome data was known for these patients.

Use of anti-reflux medication was higher in the more recent cohort (*p* < 0.001) (Table 2).

### Comparison of left and right sided defects

This comparison was exploratory. In the infants born between 2015 and 2025, there were no significant demographic differences in gestational age, birthweight or gender according to the side of defect. Preterm prelabour rupture of membranes (PPROM) was reported in 67% of left CDH versus 92% of right CDH but occurring at an earlier gestational age (GA) in left CDH infants (*p* = 0.032) (Table 3).

**Table 3:** Demographics by side of defect. Data presented as median (IQR) or *n* (%). * indicates where count < 5 and Fisher’s Exact Test used. This comparison was exploratory; the one infant with bilateral CDH was not included

	Left (*n* = 18)	Right (*n* = 13)	*p* value
GA (weeks)	34.3 (32.15–36.53)	34.9 (31.50–35.50)	0.984
BW (grams)	2279 (1918.75–2552.50)	1900 (1505.00–2779.00)	0.737
Male	15 (83)	9 (69)	0.413*
PPROM	12 (67)	12 (92)	0.191*
PPROM GA (weeks)	30.2 (28.03–33.43)	34.0 (31.22–35.23)	0.032
OE LHR	0.20 (0.15–0.22)	0.24 (0.20–0.30)	0.060
Antenatal steroids	12 (67)	8 (62)	1.0*
GA at FETO (weeks)	27.0 (26.10–27.38)	26.7 (26.6–27.3)	0.809
Balloon out at delivery	13 (72)	10 (77)	1.0*

Infants with left CDH had a longer duration of stay (*p* = 0.006) and duration of mechanical ventilation (*p* = 0.04) compared to right CDH (Table 4). Left CDH infants also had a lower O/E LHR than right CDH infants (0.20 versus 0.24, *p* = 0.06) (Table 3). There was no significant difference in mortality between the two groups (50% versus 38.5%, *p* = 0.72), but the comparison was underpowered and should not be interpreted as evidence of equivalence.

**Table 4:** Outcomes by side of defect. Data presented as median (IQR) or *n* (%). The asterisk (*) indicates where count < 5 and Fisher’s Exact Test used. In the left-sided cohort, one patient was transferred out for ECMO and in the right-sided cohort one patient transferred out for ENT review and bronchoscopy; survival data but no further outcome data was known for these patients

	Left (*n* = 18)	Right (*n* = 13)	*p* value
Age of CDH repair (days)	7.0 (4.50–8.00)	7.0 (4.50–9.00)	1.0
Proportion of infants undergoing surgical repair	10 (55.6)	9 (69.2)	0.484*
Mortality	9 (50)	5 (38.5)	0.717*
Mortality prior to repair	8 (44.4)	4 (30.8)	0.484*
Mortality post repair	1 (5.6)	1 (7.7)	1.0*
Duration MV (days)	26.5 (17.75–34.5)	16.0 (14.00–21.00)	0.04
Length of supplementary oxygen (days)	50.5 (40.50–101.25)	32.0 (28.00–39.00)	0.021
Pulmonary hypertension	6/8 (75)	4/7 (57)	0.608*
Length of stay (days)	72.0 (56.25–108.75)	44.0 (42.00–53.00)	0.006
Supplementary oxygen at discharge	2/8 (25)	0 (0)	0.467*
Age at full feeds (days)	35.0 (27.00–53.75)	32.0 (23.00–33.00)	0.189
Anti-reflux medication at discharge	7/8 (88)	6/7 (86)	1.0*
Tube feeding at discharge	2/8 (25)	4/7 (57)	0.315*
Gastrostomy	2/8 (25)	1/7 (14)	1.0*
Fundoplication	0 (0)	1/7 (14)	0.467*

## Discussion

This study demonstrated an improvement in the mortality of infants with severe CDH treated with FETO over the past two decades. This was despite the more recent cohort including a greater number of infants with right-sided defects, who historically have a worse prognosis [13]. The significant difference in O/E LHR between the two epochs (0.16 versus 0.21, *p* = 0.018) is likely due to the higher number of right sided defects, given there was no statistically significant difference in the O/E LHR of the left sided defects between eras (0.16 versus 0.19, *p* = 0.125). Changes in the management were also identified between the two epochs. Although the demographics of the two groups were broadly comparable in terms of gestational age and birth weight, the infants in the two epochs differed in several respects besides management, including side of defect, sex and antenatal steroid use, so the interpretation of the improvement cannot be taken as a proven causal effect of changes in perinatal and surgical management. Additionally, the lower bound of the 95% confidence interval for the odds ratio (1.01) lay close to the null. Three infants in the first epoch died because the FETO balloon could not be deflated. As this is a recognised, procedure-related complication of FETO, these infants were included in the mortality analysis; all of these deaths occurred in the first epoch, which may reflect the learning curve of managing these infants in a high-volume FETO centre.

The later timing of surgical repair in the recent cohort likely represents a shift towards prioritising preoperative stabilisation. This allows time for optimisation of haemodynamics and pulmonary hypertension management before repair, which is consistent with current international practice [11]. The adoption of a laparostomy at the time of CDH repair (and subsequent abdominal closure once stability has been reached), in 77.8% of recent cases marks a change in surgical strategy. A Canadian study reported rates of delayed fascial closure to be around 10% in an attempt to reduce the risk of abdominal compartment syndrome [14]. It is plausible that this contributed to the reduction in recurrence rates, from 33% to none, perhaps by minimising tension and intra-abdominal pressure on the repair site, although this remains an unproven hypothesis. It should be noted, however, that although all surviving infants undergo standardised long-term clinical follow-up, infants in the contemporary epoch had a shorter observation period and further recurrences may emerge with time. Recurrence is typically detected on routine surveillance imaging or on re-presentation with symptoms months to years after repair, so the absence of recurrence in the recent cohort may in part reflect shorter observation time rather than a true reduction in risk, particularly as patch use, a recognised risk factor for recurrence, was higher in the recent cohort (94% vs 74%).

All of the later cohort had echocardiogram-guided inotropic and ventilatory management. This was not possible in the first cohort when the use of echocardiography was more limited. Individuals with CDH have altered pulmonary vascular growth with pathological vascular remodelling and pulmonary vasoreactivity due to pulmonary hypoplasia and altered lung architecture, leading to pulmonary hypertension [15]. This can lead to right ventricular (RV) dilatation and dysfunction due to increased afterload. Additionally, there may be left ventricular (LV) dysfunction due to developmental hypoplasia of the LV or secondary to RV dysfunction and dilatation [16]. There is also a subset of infants with biventricular dysfunction. The differing clinical phenotypes require a personalised approach using echocardiography to tailor inotropic management, pulmonary vasodilators and the cautious use of nitric oxide [10].

Despite improved survival, infants in the recent era required longer mechanical ventilation and oxygen therapy, with a higher proportion remaining oxygen dependent at 28 days. This is consistent with a broader phenomenon whereby improved survival shifts the composition of the surviving cohort. Infants with more severe pulmonary hypoplasia who would previously not have survived are now included, making respiratory morbidity more visible. A longer time to complete surgical repair following abdominal wall closure may also have contributed. Although prolonged oxygen dependence could signify greater chronic respiratory morbidity, similar supplementary oxygen requirements at discharge is reassuring.

Feeding outcomes were broadly similar between the cohorts; however, the increased use of anti-reflux therapy in the contemporary group may indicate heightened clinical awareness and proactive management of gastro-oesophageal reflux, a common sequela in CDH survivors which is standard international practice [11]. Longer hospital stays in the recent cohort likely reflect greater survival of infants with severe disease, necessitating more extensive respiratory and nutritional support prior to discharge.

In both cohorts ECMO utilisation was low (0/34 [0%] in 2002–2007 versus 1/24 [4.2%] in 2020–2025). ECMO is not available on site at King’s College Hospital, therefore necessitating careful optimization of postnatal management, with referral for ECMO used as ‘rescue’ for the most critically unwell infants. While there remains ongoing debate regarding the role of ECMO in severe CDH, emerging evidence suggests that outcomes may be improved through optimised medical management and a reduction in ECMO utilisation in selected cases [17]. Nevertheless, there is a recognised role for ECMO in the management of the most severely affected infants [18, 19].

Due to severe CDH being a rare disease, the laterality comparison was exploratory and underpowered (*n* = 31), and the absence of a statistically significant difference in mortality should not be interpreted as evidence of equivalence. This study’s results agree with and provide an update to the previous literature reported by our group (which included infants born between 2004 and 2018, giving some patient overlap with the current study) and showed no significant difference in mortality between right and left-sided CDH following FETO [20]. Novelly, our results did show an increase in morbidity in the left sided defects with an increase in duration of mechanical ventilation (26.5 days versus 16.0 days, *p* = 0.040), increase in duration of supplementary oxygen (50.5 days versus 32.0 days, *p* = 0.021) and an increase in length of neonatal admission (72.0 days versus 44.0 days, *p* = 0.006). Other studies have suggested that laterality alone does not independently determine survival. Right-sided defects have been associated with a higher proportion of larger C and D defects [13], as well as increased long-term morbidity including hernia recurrence *p* = 0.043, pectus deformity *p* = 0.019, scoliosis *p* = 0.029, and chronic cough *p* = 0.001 [20]. In addition, there is evidence that left sided defects are associated with more severe left heart hypoplasia than right-sided defects of comparable severity [21], which may account for the longer need for respiratory support and length of stay experienced by those with left sided lesions. The left-sided infants, did however, have a lower O/E LHR than right-sided infants (0.20 versus 0.24, *p* = 0.06), and this difference, rather than or in addition to a distinct cardiac phenotype, may account for their greater respiratory morbidity. We did not collect echocardiographic data on ventricular size or function or on liver position, which would be needed to test the cardiac hypothesis directly. These findings are therefore hypothesis-generating, and analysing the results of right and left sided lesions separately may be beneficial.

This study has strengths and some limitations. Despite KCH being a high volume FETO centre, severe CDH infants who undergo FETO are still a rare cohort. Therefore, the numbers are relatively low limiting the ability to detect statistically significant differences in some outcomes. We acknowledge that there is just over a decade (2008–2019) of data which has been unexamined, due to the selection of the time periods used. As a result, we cannot describe the trajectory of change within this period. Furthermore, as KCH is a tertiary referral centre with over 90% of infants with CDH not originating from the local area in both eras, the majority of infants with severe CDH were referred following antenatal diagnosis elsewhere. Consequently, data on the total number of antenatally diagnosed cases including those referred for treatment and those where pregnancies were terminated were not available to us. Additionally, the follow up duration of the second epoch was necessarily shorter and may underrepresent the recurrence seen in this later cohort. Median follow-up duration was not available, and some patients in the later cohort had less than 1 year of follow-up. The laterality analysis (*n* = 31) was exploratory and was numerically dominated by the second epoch (2020–2025). Echocardiographic data on ventricular size or function and on liver position were not collected, so we could not test whether cardiac phenotype explained the differences in morbidity between left- and right-sided defects. Several comparisons yielded *p*-values close to 0.05 and multiplicity was not formally accounted for, so these results should be interpreted with caution. Differences between the epochs in side of defect, sex and antenatal steroid use may have confounded the survival comparison, and the small number of deaths (*n* = 26) did not allow a reliable adjusted analysis. Our findings derive from a single, high-volume tertiary FETO centre with integrated fetal medicine, neonatal and paediatric surgical services so they may not be generalisable to centres with lower FETO volumes, different antenatal selection or referral pathways, or on-site ECMO. Nevertheless, this study has notable strengths. This study represents one of the largest single-centre datasets of FETO-treated severe CDH infants in Europe and provides detailed neonatal outcome data over two decades. The inclusion of both surgical and respiratory outcome data allows for a comprehensive assessment of the impact of evolving management strategies. Furthermore, the analysis of outcomes by side of defect in a FETO-treated cohort adds novel findings to the existing literature, generating the hypothesis that left and right sided CDH may differ in clinical phenotype, which warrants evaluation in larger studies.

## Conclusions

The improvement in mortality seen at this high volume FETO centre over the two epochs is likely multifactorial and reflecting changes in all aspects of management. The data from this study suggest that a multidisciplinary approach to infants with severe CDH, providing personalised care in a high-volume centre is beneficial and could be associated with improved outcomes in CDH. In the contemporary cohort, left-sided defects carried greater respiratory morbidity and longer hospital stay than right-sided defects without a statistically significant difference in survival in this exploratory analysis; larger studies are needed to determine whether laterality defines distinct phenotypes.

## Data Availability

No datasets were generated or analysed during the current study.

## Abbreviations

BW: Birth weight
CDH: Congenital diaphragmatic hernia
ECMO: Extracorporeal membrane oxygenation
FETO: Fetoscopic endoluminal tracheal occlusion
GA: Gestational age
IQR: Interquartile range
KCH: King’s College Hospital
LV: Left ventricle/left ventricular
MV: Mechanical ventilation
NHS: National Health Service
O/E LHR: Observed to expected lung to head ratio
PPROM: Preterm prelabour rupture of membranes
RV: Right ventricle/right ventricular
SPSS: Statistical Package for the Social Sciences
TOTAL: Tracheal Occlusion To Accelerate Lung growth (trial)
